# Management of psychotropic medications in adults with intellectual disability: a scoping review protocol

**DOI:** 10.12688/hrbopenres.13170.1

**Published:** 2021-03-22

**Authors:** Ashley Costello, Cian Hehir, Drona Sharma, Owen Doody, Dervla Kelly

**Affiliations:** 1 School of Medicine, University of Limerick, Castletroy, Limerick, Ireland; 2 Nua Healthcare Services, Republic of Ireland, Ireland; 3 Health Research Institute, University of Limerick, Limerick, Ireland; 4 Department of Nursing and Midwifery, University of Limerick, Castletroy, Limerick, Ireland

**Keywords:** Intellectual Disability, Prescribing, De-prescribing, Psychotropic Medicine, Medication, Medication management, Scoping Review

## Abstract

**Introduction:** Psychotropic medications are commonly prescribed among adults with intellectual disability (ID), often in the absence of a psychiatric diagnosis. As such, there is great disparity between the estimated prevalence of mental illness and the rates of psychotropic medication use amongst people with ID. ‘Off-label’ use of these medications may account for much of this discrepancy, in particular their use in the management of challenging behaviour. This has come under scrutiny due to the myriad of side effects and the deficiency of high-quality data supporting their use for this indication. Understanding the causes and justifications for such disparity is essential in discerning the efficacy of current prescription practice.

**Objective**: To explore the existing evidence base regarding the prescription and management of psychotropic medications in adults with ID. The aim will be achieved through identifying the psychotropic medications commonly prescribed, the underlying rationale(s) for their prescription and the evidence available that demonstrates their appropriateness and effectiveness. Additionally, the paper will seek to evaluate the availability of any existing guidance that informs the management of these medications, and the evidence and outcomes of psychotropic medication dose reduction and/or cessation interventions.

**Inclusion criteria:** This review will consider studies that focus on the use of psychotropic medications amongst patients with ID.

**Methods: **Research studies (qualitative, quantitative and mixed design) and Grey Literature (English) will be included. The search will be conducted without time restrictions. Databases will include: Ovid MEDLINE, Embase, CINAHL, JBI Evidence Synthesis, Cochrane Central Register of Controlled Trials, Cochrane Databased of Systematic Reviews, PsycINFO and Scopus. A three-step search strategy will be followed, with results screened by two independent reviewers. Data will be extracted independently by two reviewers using a data extraction tool with results mapped and presented using a narrative form supported by tables and diagrams.

## Disclaimer

The views expressed in this article are those of the author(s). Publication in HRB Open Research does not imply endorsement by the Health Research Board of Ireland.

## Introduction

Intellectual disability (ID) is defined as a lifelong disorder that includes both intellectual and adaptive functioning deficits in conceptual, social, and practical domains, the onset of which occurs during the developmental period of life ^1^. The global prevalence of ID is estimated to be approximately 1% ^2, 3^. The reported prevalence of mental illness amongst adults with ID is inconsistent; one systematic review reported prevalence figures ranging from 3.9 to 46.3% whilst others report an even broader range that spanned from 13.9 to 75.2% ^4, 5^. It has been suggested that Diagnostic Masking (DM) and Diagnostic Overshadowing (DO) pose as significant obstacles to formal clinical diagnoses within this cohort. DM describes a clinical scenario when symptoms of mental illness are concealed or masked by pre-existing ID while DO occurs when clinicians circumscribe the diagnostic process and mislabel complex symptoms of mental illness as manifestations of ID ^6^. It has been identified that people with ID are faced with challenges in gaining access to psychiatric healthcare and support ^7, 8^. Furthermore, atypical clinical presentations of psychiatric illness, along with communication and health literacy barriers may contribute to an overall underestimation of prevalence of mental illness in the ID population ^3^. However, despite the disparity in reported prevalence, coexisting mental illness is suggested to be more prevalent in people with ID compared to the general population ^4, 5, 9^.

Psychotropic medications are commonly prescribed amongst adults and older adults with ID. For the purpose of this scoping review, the four major classes of psychotropics we will focus on are antipsychotics, antidepressants, anxiolytics and mood-stabilisers, which include lithium and anti-epileptics with mood stabilising indications. Although many of these medications indeed have indications for the management of mental illness, research has indicated poor correlation between the prescription rates of these medications and the rates of diagnosed mental illness in the ID population ^10, 11^. This discrepancy has been attributed to the ‘off-label’ use of psychotropic medication for the management of challenging behaviour, which is an unauthorised indication. Challenging behaviour encompasses behaviours of a destructive nature, such as aggression, violence and self-injury ^12– 14^. It is recognised that people with ID are at a higher risk of exhibiting challenging behaviour; the prevalence of which is typically quoted between 10 and 15% ^12, 15^. According to the National Institute for Health & Care Excellence (NICE) guidance, rates of challenging behaviour are higher in the early 20’s age group and can be as high as 30–40% in hospital settings ^14^. As a consequence, the ID patient cohort are at increased risk for prescription of psychotropic medications not only for the management of mental illness, but also for the treatment of challenging behaviour, in the absence of a psychiatric diagnosis. In fact, research carried out in the United Kingdom (UK) and North America has suggested that challenging behaviour is one of the most common reasons for the prescription of psychotropic drugs ^16^. Despite their widespread usage, there exists a dearth of high quality data available to inform the provision of these medications in this patient subgroup ^17, 18^.

Concerns regarding the prescription of these medications within the ID patient population have been raised over the years ^3^. Psychotropic medications are associated with a myriad of risks ranging from metabolic and hormonal dysfunction to extrapyramidal side effects that can adversely affect movement. They are also associated with cardiovascular side effects such as arrhythmias and QT-interval prolongation, hyperglycaemia and weight gain, along with the risk of potentially fatal neuroleptic malignant syndrome ^19– 21^. Such a combination of side effects becomes increasingly concerning considering a higher prevalence of significant comorbidities, lessened seizure thresholds and a reduced capacity to self-report adverse effects within this highly vulnerable patient group ^22^. Furthermore, people with ID are considered to be at an elevated risk for developing said side effects ^23^.

NICE advises implementation of psychological and environmental interventions for the management of challenging behaviour as the first step and recommends the consideration of psychotropic medication only in particular circumstances; for example, when there is a severe risk to the person or others ^14^. These guidelines recommend the continuation of these medication on the basis of a beneficial response. With this in mind, it would seem that to achieve reduction and/or cessation of these medications would be a desirable outcome. However, due to the paucity of research regarding psychotropic discontinuation, people with ID tend to be treated for prolonged periods of time despite the associated risks. What is more, the challenging behaviour for which psychotropics are frequently prescribed to manage often remains unchanged ^24, 25^.

Despite a small body of research on this topic, the advice to health care professionals remains unclear. Hence, the aim of this scoping review is to investigate the literature available on the use of psychotropic medications within the ID cohort. This scoping review aims to identify what psychotropic medications are prescribed to adults with ID, why they are prescribed to this patient cohort and how these medications are managed. This will be carried out with special focus on interventions that aim to achieve dose reduction or complete cessation of psychotropics and to identify the associated risks and benefits that accompany this reduction/cessation. This scoping review will assist to identify any gaps in the literature available and to help guide and recommend future studies and systematic literature reviews within this area of research. 

## Research questions (RQs)

1) What psychotropic medications are commonly prescribed among adults with ID?
2) What is the clinical indication(s) for prescription of such medications?
3) What evidence base (if any) exists to support the prescription of psychotropic medications, including ‘off-label’ use in adults with ID?
4) What guidelines/policies exist regarding the management of psychotropic medicines once they are prescribed among people with ID?
5) What interventions (if any) are available to facilitate dose reduction or cessation of psychotropic medications among people with ID?
  - How have such interventions been evaluated to date? i.e. what outcomes are measured?
  - What are the potential benefits and risks associated with the reduction or cessation of psychotropic medication?

## Methods

The protocol was drafted according to the Preferred Reporting Items for Systematic Reviews and Meta-analyses extension for Scoping Reviews (PRISMA-ScR) protocol ^26^.

### Inclusion and exclusion criteria

Inclusion criteria

- Participants: all adults (>18 years of age) with ID, regardless of demographic or clinical characteristics.
- Concept: interventions and/or phenomena of interest (reduction/cessation of psychotropic meds in adults with ID).
- Outcomes:
  - Any qualitative or quantitative outcome reporting on psychotropic medication use, practices or behaviours among ID population their carers or their prescribers.
  - Any qualitative or quantitative outcome reporting on psychotropic medication safety measures (adverse drug event, adverse drug reaction, medication error, adherence, compliance, consumption, drug-related problems).
- Study design: all research designs including reviews (systematic, integrative and narrative) and research (qualitative, quantitative and mixed design studies). In addition, national and international policies, strategies, guidelines and standards will also be examined.
- Year of publication: No restriction.
- Language: No restriction.

Considering the small body of research available on this topic, broad inclusion criteria were developed to ensure all relevant research is captured whilst reducing the risk of omissions. 

Exclusion criteria

- Article types: commentaries, editorials, opinion pieces, non-systematic literature reviews.
- Clinical trials of medicinal products.

### Search

The proposed scoping review search will begin in December 2020 and continue throughout January and February 2021. The search will be conducted according to the three steps of Joanna Briggs Institute (JBI) methodology for scoping reviews:

1. The CINAHL and PsychInfo databases were initially searched to identify papers on the topic. The search terms used for this initial search are provided as *Extended data* (Table 1) ^27^. Text words contained in the titles and abstract of included articles and within the index terms (describing the articles) were used to develop a full search strategy for CINAHL complete database (Table 2, *Extended data* ^27^). This search strategy (including its identified keywords and index terms) will be adapted for all the information sources included in this scoping review.
2. A second search will be undertaken across all included databases, namely: Ovid MEDLINE, Embase, CINAHL, JBI Evidence Synthesis, Cochrane Central Register of Controlled Trials, Cochrane Database of Systematic Reviews, PsycINFO and Scopus. This will be done using all the identified keywords and index terms. Grey literature databases will also be searched (Open Grey, reports, dissertations, theses databases and databases of conference abstracts (e.g. Scopus (for conference proceedings only), ETHOS, ProQuest) for national and international strategies and policies as well as standards and guidance documents.
3. The reference lists of the articles and reports identified and included in the review will be searched for further studies. If warranted, authors of included articles will be contacted for further information.

### Evidence selection

The selection of evidence to be included in the scoping review will be carried out independently by two reviewers. Following the search, all identified records will be reviewed and duplicates excluded. Thereafter, titles and abstracts will be assessed for inclusion. The remaining studies full texts will be screened against the inclusion criteria and the reasons for exclusion will be identified and recorded. This process will be carried out using the reference management software ‘Rayyan’ ^28^. Any discrepancies that may arise regarding evidence selection will be resolved through discussion and consensus with a third reviewer.

### Data extraction and reporting

Data will be extracted independently by two reviewers, conflicts resolved by consensus or discussion with a third reviewer. A data extraction tool developed by the reviewers will be piloted in four studies by the reviewers working in pairs. The review pairs will discuss the usability of the tool, any possible additions or changes in order to evaluate and/or modify the tool prior to adoption. Any adaptations to the tool will be documented clearly. Thereafter, the data extraction tool will be utilised independently by the two reviewers during appraisal of the evidence base.

The data extraction tool will include the following details:

- Names of the authors, year of publication, country of origin,
- Medication usage: prevalence, types, indications, dosage, duration of use, setting (RQ1 and RQ2)
- Medication effectiveness: clinical effectiveness measures, side effects, drug interactions, experiences of patients (RQ3)
- Medication management intervention designs: population, type of intervention, any comparator and setting, healthcare professionals involved (RQ4 and RQ5)
- Outcomes of medication management programs (RQ4 and RQ5)

Reporting of key information from the chosen studies will be performed using the data extraction tool provided as *Extended data* (Table 3) ^27^.

### Data presentation

The results will be mapped and presented in relation to each of the research questions. The results of the review will be presented in a narrative form. As necessary, tables and diagrams will be utilized to illustrate findings augmented by narrative text. Results will be reported and presented in accordance with PRISMA-ScR reporting guidance ^26^ and the PRISMA flow diagram ^29^.

## Study status

The search in currently underway across databases outlined in methods section. This search will take place from December 2020 and continue throughout January and February 2021.

## Data availability

### Underlying data

No underlying data are associated with this article.

### Extended data

Zenodo: Management of psychotropic medications in adults with intellectual disability: a scoping review protocol. https://doi.org/10.5281/zenodo.4446015 ^27^.

This project contains the following extended data in the document ‘Extended Data.docx’:

- Table 1: Preliminary Search
- Table 2: Full search strategy
- Table 3: Data extraction tool

Data are available under the terms of the Creative Commons Attribution 4.0 International license (CC-BY 4.0).

## References

[1] American Psychiatric Association: Diagnostic and Statistical Manual of Mental Disorders.Diagnostic and Statistical Manual of Mental Disorders FEA, VA: American Psychiatric Association,2013. Reference Source

[2] Maulik PK Mascarenhas MN Mathers CD : Prevalence of intellectual disability: a meta-analysis of population-based studies. *Res Dev Disabil.* 2011;32(2):419–36. 10.1016/j.ridd.2010.12.018 21236634

[3] Sheehan R Hassiotis A Walters K : Mental illness, challenging behaviour, and psychotropic drug prescribing in people with intellectual disability: UK population based cohort study. *BMJ.* 2015;351: h4326. 10.1136/bmj.h4326 26330451 PMC4556752

[4] Whitaker S Read S : The Prevalence of Psychiatric Disorders among People with Intellectual Disabilities: An Analysis of the Literature. *J Appl Res Intellect Disabil.* 2006;19(4):330–345. 10.1111/j.1468-3148.2006.00293.x

[5] Buckles J Luckasson R Keefe E : A Systematic Review of the Prevalence of Psychiatric Disorders in Adults With Intellectual Disability, 2003–2010. *J Ment Health Res Intellect Disabil.* 2013;6(3):181–207. 10.1080/19315864.2011.651682

[6] Manohar H Subramanian K Kandasamy P : Diagnostic Masking and Overshadowing in Intellectual Disability-How Structured Evaluation Helps. *J Child Adolesc Psychiatr Nurs.* 2016;29(4):171–176. 10.1111/jcap.12160 27901303

[7] McCarthy J Boyd J : Mental health services and young people with intellectual disability: is it time to do better? *J Intellect Disabil Res.* 2002;46(Pt 3):250–6. 10.1046/j.1365-2788.2002.00401.x 11896810

[8] Gulliford M Figueroa-Munoz J Morgan M : What does 'access to health care' mean? *J Health Serv Res Policy.* 2002;7(3):186–8. 10.1258/135581902760082517 12171751

[9] Ahlstrom G Axmon A Sandberg M : Specialist psychiatric health care utilization among older people with intellectual disability - predictors and comparisons with the general population: a national register study. *BMC Psychiatry.* 2020;20(1):70. 10.1186/s12888-020-02491-6 32066421 PMC7027029

[10] Perry BI Cooray SE Mendis J : Problem behaviours and psychotropic medication use in intellectual disability: a multinational cross-sectional survey. *J Intellect Disabil Res.* 2018;62(2):140–149. 10.1111/jir.12471 29349928

[11] Robertson J Emerson E Gregory N : Receipt of psychotropic medication by people with intellectual disability in residential settings. *J Intellect Disabil Res.* 2000;44(Pt 6):666–76. 10.1111/j.1365-2788.2000.00307.x 11115021

[12] Tanwar M Lloyd B Julies P : Challenging behaviour and learning disabilities: prevention and interventions for children with learning disabilities whose behaviour challenges: NICE guideline 2015. *Arch Dis Child Educ Pract Ed.* 2017;102(1):24–27. 10.1136/archdischild-2015-309575 27707756

[13] Deutsch SI Burket JA : Psychotropic medication use for adults and older adults with intellectual disability; selective review, recommendations and future directions. *Prog Neuropsychopharmacol Biol Psychiatry.* 2021;104:110017. 10.1016/j.pnpbp.2020.110017 32544599

[14] Tong M Longato L de la Monte SM : Early limited nitrosamine exposures exacerbate high fat diet-mediated type 2 diabetes and neurodegeneration. *BMC Endocr Disord.* 2010;10:4. 10.1186/1472-6823-10-4 20302640 PMC3161394

[15] Emerson E Kiernan C Alborz A : The prevalence of challenging behaviors: a total population study. *Res Dev Disabil.* 2001;22(1):77–93. 10.1016/s0891-4222(00)00061-5 11263632

[16] Molyneux P Emerson E Caine A : Prescription of Psychotropic Medication to People with Intellectual Disabilities in Primary Health‐care Settings. *J Appl Res Intellect Disabil.* 1999;12(1):46–57. 10.1111/j.1468-3148.1999.tb00049.x

[17] Deb S Sohanpal SK Soni R : The effectiveness of antipsychotic medication in the management of behaviour problems in adults with intellectual disabilities. *J Intellect Disabil Res.* 2007;51(Pt 10):766–77. 10.1111/j.1365-2788.2007.00950.x 17803495

[18] Matson JL Neal D : Psychotropic medication use for challenging behaviors in persons with intellectual disabilities: an overview. *Res Dev Disabil.* 2009;30(3):572–86. 10.1016/j.ridd.2008.08.007 18845418

[19] de Leon J Greenlee B Barber J : Practical guidelines for the use of new generation antipsychotic drugs (except clozapine) in adult individuals with intellectual disabilities. *Res Dev Disabil.* 2009;30(4):613–69. 10.1016/j.ridd.2008.10.010 19084370

[20] Sheehan R Hassiotis A : Reduction or discontinuation of antipsychotics for challenging behaviour in adults with intellectual disability: a systematic review. *Lancet Psychiatry.* 2017;4(3):238–256. 10.1016/S2215-0366(16)30191-2 27838214

[21] Emerson E : Challenging Behaviour: Analysis and Intervention in People with Learning Difficulties.Cambridge University Press, Cambridge.1995. Reference Source

[22] Sullivan WF Berg JM Bradley E : Primary care of adults with developmental disabilities: Canadian consensus guidelines. *Can Fam Physician.* 2011;57(5):541–53. 21571716 PMC3093586

[23] Shuker DE Tannenbaum SR : Determination of nonvolatile N-nitroso compounds in biological fluids by liquid chromatography with postcolumn photohydrolysis detection. *Anal Chem.* 1983;55(13):2152–5. 10.1021/ac00263a034 6650839

[24] Marshall T : Audit of the use of psychotropic medication for challenging behaviour in a community learning disability service. *Psychiatric Bulletin.* 2004;28(12):447–450. 10.1192/pb.28.12.447

[25] Deb S Unwin G Deb T : Characteristics and the trajectory of psychotropic medication use in general and antipsychotics in particular among adults with an intellectual disability who exhibit aggressive behaviour. *J Intellect Disabil Res.* 2015;59(1):11–25. 10.1111/jir.12119 24450426

[26] Tricco AC Lillie E Zarin W : PRISMA Extension for Scoping Reviews (PRISMA-ScR): Checklist and Explanation. *Ann Intern Med.* 2018;169(7):467–473. 10.7326/M18-0850 30178033

[27] Costello A Hehir C Sharma D : Management of psychotropic medications in adults with intellectual disability: a scoping review protocol.2021. 10.5281/zenodo.4446015 PMC8503790 34693205

[28] Ouzzani M Hammady H Fedorowicz Z : Rayyan-a web and mobile app for systematic reviews. *Syst Rev.* 2016;5(1):210. 10.1186/s13643-016-0384-4 27919275 PMC5139140

[29] Moher D Liberati A Tetzlaff J : Preferred Reporting Items for Systematic Reviews and Meta-Analyses: The PRISMA statement. *PLoS Med.* 2009;6(7): e1000097. 10.1371/journal.pmed.1000097 19621072 PMC2707599

