# Supplementary material for: Management of psychotropic medications in adults with intellectual disability: a scoping review protocol
Source: HRB Open Res. 2022 Jan 12;4:30. Originally published 2021 Mar 22. [Version 2] doi: 10.12688/hrbopenres.13170.2 (PMC8503790; doi:10.12688/hrbopenres.13170.2)
Supplement: Peer review history

# Peer review: Reviewer response for version 2

**DOI:** 10.21956/hrbopenres.14691.r31228

**Published:** 2023-06-19

**Authors:** David Harley

**Affiliations:** 1 UQ Centre for Clinical Research, The University of Queensland, Herston, Qld, Australia

The authors have responded in full to comments from two other reviewers and me. I am satisfied with their responses.

Is the study design appropriate for the research question?

Yes

Is the rationale for, and objectives of, the study clearly described?

Yes

Are sufficient details of the methods provided to allow replication by others?

Partly

Are the datasets clearly presented in a useable and accessible format?

Not applicable

Reviewer Expertise:

Adult developmental disability medicine, with a particular interest in psychotropic prescribing.

I confirm that I have read this submission and believe that I have an appropriate level of expertise to confirm that it is of an acceptable scientific standard.

# Peer review: Reviewer response for version 1

**DOI:** 10.21956/hrbopenres.14295.r30244

**Published:** 2021-10-08

**Authors:** David Harley

**Affiliations:** 1 UQ Centre for Clinical Research, The University of Queensland, Herston, Qld, Australia

This is a well-written protocol for a study addressing a very important topic. The article describes the methods for a scoping review of the use of psychotropic medications in people with intellectual disabilities. The authors' aim is “To explore the existing evidence base regarding the prescription and management of psychotropic medications in adults with ID”. The proposed review will encompass the types of drugs prescribed, indications and evidence base, guidelines for management, and protocols for cessation. The topic chosen is important because the health of people with an intellectual disability is poor and because the inappropriate use of psychotropic medications significantly contributes to this poor health status. In addition, clinicians who manage people with intellectual disabilities need evidence for their prescribing, and this review has the potential to significantly contribute here.

 The scope for the review is quite broad. It would be good to have more information on words to be used in the search because this would allow replication. Important context for this work is the definition of challenging behaviour along with its precipitants. This is because prescribing, and particularly inappropriate prescribing, is often a response to challenging behaviour. One of the research questions is, “What is the clinical indication(s) for prescription of such medications?", and it is well recognised that challenging behaviour is often cause for prescribing. An acknowledgement, because this relates to some of the research questions, of the myriad precipitants for challenging behaviour is crucial. Challenging behaviour is a means of communication, and many of the causes, such as sexual abuse, constipation, and pain, are not appropriately managed with psychotropic medications.

Is the study design appropriate for the research question?

Yes

Is the rationale for, and objectives of, the study clearly described?

Yes

Are sufficient details of the methods provided to allow replication by others?

Partly

Are the datasets clearly presented in a useable and accessible format?

Not applicable

Reviewer Expertise:

Adult developmental disability medicine, with a particular interest in psychotropic prescribing.

I confirm that I have read this submission and believe that I have an appropriate level of expertise to confirm that it is of an acceptable scientific standard, however I have significant reservations, as outlined above.

# Response

**Published:** 2021-12-03

**Authors:** Ashley Costello

**Affiliations:** University of Limerick, Ireland

Dear Editor and Colleagues,

 Thank you all for reviewing our paper. Your comments have been very helpful. We appreciate the opportunity to respond and have addressed all comments, point-by-point.

**Reviewer 3:**

 David Harley Peer Review (Approved with Reservations)

**Reviewer:** This is a well-written protocol for a study addressing a very important topic. The article describes the methods for a scoping review of the use of psychotropic medications in people with intellectual disabilities. The authors' aim is “To explore the existing evidence base regarding the prescription and management of psychotropic medications in adults with ID”. The proposed review will encompass the types of drugs prescribed, indications and evidence base, guidelines for management, and protocols for cessation. The topic chosen is important because the health of people with an intellectual disability is poor and because the inappropriate use of psychotropic medications significantly contributes to this poor health status. In addition, clinicians who manage people with intellectual disabilities need evidence for their prescribing, and this review has the potential to significantly contribute here.

**Reviewer:** The scope for the review is quite broad. It would be good to have more information on words to be used in the search because this would allow replication.

**Response: **The search terms are provided in Table 1 in the extended data.

**Reviewer:** Important context for this work is the definition of challenging behaviour along with its precipitants. This is because prescribing, and particularly inappropriate prescribing, is often a response to challenging behaviour. One of the research questions is, “What is the clinical indication(s) for prescription of such medications?", and it is well recognised that challenging behaviour is often cause for prescribing. An acknowledgement, because this relates to some of the research questions, of the myriad precipitants for challenging behaviour is crucial. Challenging behaviour is a means of communication, and many of the causes, such as sexual abuse, constipation, and pain, are not appropriately managed with psychotropic medications.

**Response: **We have revised our definition of challenging behaviour in the introduction to as follows: “As defined by The Royal College of Psychiatrists, challenging behaviour is behaviour of such an intensity, frequency or duration as to threaten the quality of life and/or the physical safety of the individual or others and is likely to lead to responses that are restrictive, aversive or result in exclusion (13). It may include behaviours of a destructive nature, such as aggression, violence and self-injury (14-16). Challenging behaviour can also be an attempt to communicate unmet needs, which require identification of causes and promotion of positive behaviours and addressing social needs.”

# Peer review: Reviewer response for version 1

**DOI:** 10.21956/hrbopenres.14295.r30254

**Published:** 2021-09-13

**Authors:** Ashok Roy

**Affiliations:** 1 University of Warwick, Coventry, UK; 2 Health Education England, London, UK

This scoping review aims to investigate available literature, find gaps, and recommend future studies and systematic reviews. Additional keywords may include "overmedication" and STOMP (Stopping Overmedication in People with Learning Disability), a national initiative in the UK that has been the subject of recent research and audit.

 The authors are looking for outcome reporting on "practices or behaviours among ID population, their carers or their prescribers". This is very broad. It would be clearer if the term "behaviours" was used in relation to the ID population and "practices" for prescribers or even non-prescribing professionals. This specific issue is not covered by the Research Questions.

 The protocol is clearly laid out and the references are relevant and recent. I found *Systematic review or scoping review? Guidance for authors when choosing between a systematic or scoping review approach* by Munn, Z. *et al*. BMC Medical Research Methodology 18, 143 (2018) ^1^ useful****** to understand why the authors had opted for carrying out a scoping review rather than carrying out a systematic review.

Is the study design appropriate for the research question?

Yes

Is the rationale for, and objectives of, the study clearly described?

Yes

Are sufficient details of the methods provided to allow replication by others?

Yes

Are the datasets clearly presented in a useable and accessible format?

Yes

Reviewer Expertise:

Intellectual Disability Psychiatry

I confirm that I have read this submission and believe that I have an appropriate level of expertise to confirm that it is of an acceptable scientific standard.

## References

[1] : Systematic review or scoping review? Guidance for authors when choosing between a systematic or scoping review approach. *BMC Medical Research Methodology* .2018;18(1) : 10.1186/s12874-018-0611-x 10.1186/s12874-018-0611-x 30453902PMC6245623

# Response

**Published:** 2021-12-03

**Authors:** Ashley Costello

**Affiliations:** University of Limerick, Ireland

Dear Editor and Colleagues,

 Thank you all for reviewing our paper. Your comments have been very helpful. We appreciate the opportunity to respond and have addressed all comments, point-by-point.

**Reviewer 2**

 Ashok Roy Peer Review (Approved)

**Reviewer:** This scoping review aims to investigate available literature, find gaps, and recommend future studies and systematic reviews. Additional keywords may include "overmedication" and STOMP (Stopping Overmedication in People with Learning Disability), a national initiative in the UK that has been the subject of recent research and audit.

**Response: **Thank you. We have added these keywords to our search.

**Reviewer:** The authors are looking for outcome reporting on "practices or behaviours among ID population, their carers or their prescribers". This is very broad. It would be clearer if the term "behaviours" was used in relation to the ID population and "practices" for prescribers or even non-prescribing professionals. This specific issue is not covered by the Research Questions.

**Response:** We have now amended our outcomes to reflect this suggestion;

 “- Any qualitative or quantitative outcome reporting on psychotropic medication use and behaviours among the population with ID.

 - Any qualitative or quantitative outcome reporting on psychotropic medication safety measures (adverse drug event, adverse drug reaction, medication error, adherence, compliance, consumption, drug-related problems)

 - Any professional practices by health care providers in relation to managing psychotropics in the population with ID”

**Reviewer:** The protocol is clearly laid out and the references are relevant and recent. I found Systematic review or scoping review? Guidance for authors when choosing between a systematic or scoping review approach by Munn, Z. et al. BMC Medical Research Methodology 18, 143 (2018) 1 useful to understand why the authors had opted for carrying out a scoping review rather than carrying out a systematic review.

**Response: **We have added further justification for the scoping review to the introduction as follows: “As we are also interested in dose reductions of psychotropic medications and any accompanying psychological or social educational intervention components for challenging behaviours, we choose to undertake a scoping review rather than a systematic review to include de-prescribing studies and heterogeneous study methodologies.”

# Peer review: Reviewer response for version 1

**DOI:** 10.21956/hrbopenres.14295.r29800

**Published:** 2021-09-06

**Authors:** Lotte Ramerman

**Affiliations:** 1 Netherlands Institute for Health Services Research, Utrecht, The Netherlands

The scoping review is focused on collecting and reviewing available literature on the subject of prescribing psychotropic medications to people with ID, including for mental illness and challenging behaviors. Furthermore, it will search for literature on managing the medication and strategies to discontinue.  

 The authors provided a clearly written protocol on a very important topic. A clear description of all available knowledge will be very valuable.

**Some general remarks**

- I would suggest not using terms as ID patient but stick to “people with ID”.
- I would suggest using fewer abbreviations to improve readability. Also, check the text for not intentional abbreviations, such as “meds”.

**Rationale and objectives**

- I feel some of the review has been done (partly) before, but some time ago. Maybe the authors can refer to this and state that they are providing an update. For example, Deb, S., & Unwin, G. L. (2007). Psychotropic medication for behaviour problems in people with intellectual disability: A review of the current literature. Current Opinion in Psychiatry, 20(5), 461-466 ^1^, or the systematic reviews by Brylewski and Duggan ^2^.
- The description of challenging behavior is kind of narrow, focusing on aggression and self-injury. This does not consider other challenging behaviors such as stereotypical behaviors, withdrawn behavior, or lethargy. Challenging behavior may be defined as behavior of such an intensity, duration, or frequency that the behavior is a danger to the physical safety of the person or others. Furthermore, these behaviors could lead to exclusion from the community.
- Furthermore, a description of the underlying issues that are related to challenging behaviors would be useful. These can be mental illnesses, but can also be related to physical complaints, problems in the environment of the person, etc.
- “Psychotropic medications are commonly prescribed amongst adults and older adults with ID” needs a reference. Furthermore, psychotropic medications are not just prescribed to adults with ID, but also to children and adolescents. Why did you specifically focus on adults and older adults?
- Statements in the introduction, such as “However, due to the paucity of research regarding psychotropic discontinuation”, are a little too strong as there is research available, especially on the discontinuation of antipsychotic drugs. Furthermore, how does this statement relate to your objective in which you aim to review studies on this topic?
- Another example: “Despite a small body of research on this topic, the advice to health care professionals remains unclear”. This is much more nuanced. There are guidelines on prescribing and discontinuation of psychotropic drugs and there is research available. However, the situation of people with ID and challenging behaviors who use psychotropic drugs is often very complex.
- The focus on mental illness and/or challenging behavior remains unclear from the objective. Maybe the authors could be a little more explicit in their objectives.

**Study design**

- Why did the authors decide on a scoping review and not a systematic review?

**Methods**

- I am not familiar with the “three steps of Joanna Briggs Institute (JBI) methodology for scoping reviews”. Can you provide a reference?
- A data extraction tool developed by the reviewers is mentioned, including that it is piloted in four studies. However, it remains unclear what the relation is between the pilot and the scoping review. And, the tool needs references.
- The description of the data extraction tool in the article does not include a description of the type of studies and the quality of the studies reviewed. However, Table 3 does. A little more information on how literature is reviewed and included in the reporting would be useful.
- Furthermore, check for double information.

Is the study design appropriate for the research question?

Partly

Is the rationale for, and objectives of, the study clearly described?

Partly

Are sufficient details of the methods provided to allow replication by others?

Yes

Are the datasets clearly presented in a useable and accessible format?

Not applicable

Reviewer Expertise:

Health Services Research, People with Intellectual Disabilities, Antipsychotic Drugs, Primary (out-of hours) Care

I confirm that I have read this submission and believe that I have an appropriate level of expertise to confirm that it is of an acceptable scientific standard, however I have significant reservations, as outlined above.

## References

[1] : Psychotropic medication for behaviour problems in people with intellectual disability: a review of the current literature. *Curr Opin Psychiatry* .2007;20(5) : 10.1097/YCO.0b013e3282ab9952 461-6 10.1097/YCO.0b013e3282ab9952 17762588

[2] : Antipsychotic medication for challenging behaviour in people with learning disability. *Cochrane Database Syst Rev* .2004; 10.1002/14651858.CD000377.pub2 CD000377 10.1002/14651858.CD000377.pub2 15266428PMC12334078

# Response

**Published:** 2021-12-03

**Authors:** Ashley Costello

**Affiliations:** University of Limerick, Ireland

Dear Editor and Colleagues,

 Thank you all for reviewing our paper. Your comments have been very helpful. We appreciate the opportunity to respond and have addressed all of your comments below, point-by-point.

**Reviewer 1**

 Lotte Ramerman Peer Review (Approved with reservations)

 The scoping review is focused on collecting and reviewing available literature on the subject of prescribing psychotropic medications to people with ID, including for mental illness and challenging behaviors. Furthermore, it will search for literature on managing the medication and strategies to discontinue.  

 The authors provided a clearly written protocol on a very important topic. A clear description of all available knowledge will be very valuable.

*Some general remarks*

**Reviewer:** I would suggest not using terms as ID patient but stick to “people with ID”.

**Response: **Many thanks for highlighting this to us – edits have been made to adopt this suggestion throughout our protocol.

**Reviewer:** I would suggest using fewer abbreviations to improve readability. Also, check the text for not intentional abbreviations, such as “meds”.

**Response:** We have since removed unnecessary abbreviations and have corrected any unintentional use of “meds”.

*Rationale and objectives*

**Reviewer: **I feel some of the review has been done (partly) before, but some time ago. Maybe the authors can refer to this and state that they are providing an update. For example, Deb, S., & Unwin, G. L. (2007). Psychotropic medication for behaviour problems in people with intellectual disability: A review of the current literature. Current Opinion in Psychiatry, 20(5), 461-466 1, or the systematic reviews by Brylewski and Duggan 2.

**Response: **We have now included reference to this paper in our introduction and one other relevant review that we are aware of.

**Reviewer:** The description of challenging behavior is kind of narrow, focusing on aggression and self-injury. This does not consider other challenging behaviors such as stereotypical behaviors, withdrawn behavior, or lethargy. Challenging behavior may be defined as behavior of such an intensity, duration, or frequency that the behavior is a danger to the physical safety of the person or others. Furthermore, these behaviors could lead to exclusion from the community.

 Furthermore, a description of the underlying issues that are related to challenging behaviors would be useful. These can be mental illnesses, but can also be related to physical complaints, problems in the environment of the person, etc.

**Response: **Thank you for this helpful suggestion. We have amended our previous definition to a broader, more descriptive definition of challenging behaviour.

**Reviewer:** “Psychotropic medications are commonly prescribed amongst adults and older adults with ID” needs a reference. Furthermore, psychotropic medications are not just prescribed to adults with ID, but also to children and adolescents. Why did you specifically focus on adults and older adults?

**Response: **Our plan is to develop an intervention targeting the adult population with ID in the first instance. For that reason, our search focuses on adults. Our future research intends to broaden this to the children and adolescent population.

**Reviewer:** Statements in the introduction, such as “However, due to the paucity of research regarding psychotropic discontinuation”, are a little too strong as there is research available, especially on the discontinuation of antipsychotic drugs. Furthermore, how does this statement relate to your objective in which you aim to review studies on this topic?

 Another example: “Despite a small body of research on this topic, the advice to health care professionals remains unclear”. This is much more nuanced. There are guidelines on prescribing and discontinuation of psychotropic drugs and there is research available. However, the situation of people with ID and challenging behaviors who use psychotropic drugs is often very complex.

 The focus on mental illness and/or challenging behavior remains unclear from the objective. Maybe the authors could be a little more explicit in their objectives.

**Response: **We have edited our study aim to more explicitly illustrate the aim of the scoping review and objectives:

 “This aim of this scoping review is to investigate the existing literature on the use of psychotropic medications within the ID cohort to manage challenging behaviour”.

 “In particular, this review aims to identify what psychotropic medications are prescribed to adults with ID, why they are prescribed to this patient cohort and how these medications are managed over the long term. This will be carried out by including interventions that aim to achieve dose reduction or complete cessation of psychotropics and to identify the associated risks and benefits that accompany this reduction/cessation. As we are also interested in dose reductions of psychotropic medications and any accompanying psychological or social educational intervention components for challenging behaviours, we choose to undertake a scoping review rather than a systematic review to include de-prescribing studies.”

*Study design*

**Reviewer: **Why did the authors decide on a scoping review and not a systematic review?

**Response: **We have added further justification for the scoping review to the introduction as follows:

 “As we are also interested in dose reductions of psychotropic medications and any accompanying psychological or social educational intervention components for challenging behaviours, we choose to undertake a scoping review rather than a systematic review to include de-prescribing studies and heterogeneous study methodologies.”

*Methods*

**Reviewer:** I am not familiar with the “three steps of Joanna Briggs Institute (JBI) methodology for scoping reviews”. Can you provide a reference?

**Response: **We have now included the following reference in our protocol:

 Peters MDJ, Godfrey C, McInerney P, Munn Z, Tricco AC, Khalil, H. Chapter 11: Scoping Reviews (2020 version). In: Aromataris E, Munn Z (Editors). *JBI Manual for Evidence Synthesis*, JBI, 2020. Available from https://synthesismanual.jbi.global. https://doi.org/10.46658/JBIMES-20-12

**Reviewer:** A data extraction tool developed by the reviewers is mentioned, including that it is piloted in four studies. However, it remains unclear what the relation is between the pilot and the scoping review. And, the tool needs references.

**Response: **We have added references to the tool and explained the term ‘4 pilot studies’. “A data extraction tool developed by the reviewers will be piloted using four studies from a preliminary literature search on the research topic (Table 3, Extended data)”.

**Reviewer:** The description of the data extraction tool in the article does not include a description of the type of studies and the quality of the studies reviewed. However, Table 3 does. A little more information on how literature is reviewed and included in the reporting would be useful.

**Response: **We will chart the study components and then map them to the research questions. The charting will include the methodology and limitations of the studies, which will allow us to comment on methodological strengths and weaknesses of the existing body of research. We have added some more detail to the ‘Data extraction and reporting section’:

**“**The chart data will detail the aim of study, methodology, intervention, outcomes, findings and limitations. We will use the data to describe the context of studies selected, how relevant outcomes were measured and any reported limitations or quality issues.”
